# Minimally important difference of the Treatment Satisfaction with Medicines Questionnaire (SATMED-Q)

**DOI:** 10.1186/1471-2288-11-142

**Published:** 2011-10-20

**Authors:** Javier Rejas, Miguel A Ruiz, Antonio Pardo, Javier Soto

**Affiliations:** 1 Health Outcomes Research Department, Corporate Affairs and Market Access Unit, Pfizer España, Alcobendas (Madrid), Spain; 2 Department of Methodology, School of Psychology, Universidad Autónoma de Madrid, Madrid, Spain

**Keywords:** meaningful difference, minimally important difference, SATMED-Q, satisfaction, chronic health conditions, medicines

## Abstract

**Background:**

A previous study has documented the reliability and validity of the Treatment Satisfaction with Medicines Questionnaire (SATMED-Q) in exploring patient satisfaction with medicines for chronic health conditions in routine medical practice, but the minimally important difference (MID) of this tool is as yet unknown. The objective of this research was to estimate the MID for the SATMED-Q total score and six constituent domains.

**Methods:**

The sample of patients (456 subjects, mean age 59 years, 53% male) used for testing psychometric properties was also used to assess MID. Item #14 of the Treatment Satisfaction Questionnaire for Medication (TSQM) was used as an anchor reference since it directly explores satisfaction with medicine on a 7-point ordinal scale (from extremely satisfied to extremely dissatisfied, with a neutral category). Patients were classified into four categories according to responses to this item (extremely satisfied/dissatisfied, very satisfied/dissatisfied, satisfied/dissatisfied, neither satisfied nor dissatisfied (neutral), and calculations were made for the total score and each domain of the SATMED-Q using standardised scores. The mean absolute differences in total score (and domains) between the neutral category and the satisfied/dissatisfied category were considered to be the MID. Effect sizes (ES) were also computed.

**Results:**

The MID for the total score was 13.4 (ES = 0.91), while the domain values ranged from 10.3 (medical care domain, ES = 0.43) to 20.6 (impact on daily living, ES = 0.85). Mean differences in satisfaction (as measured by the total SATMED-Q score and domain scores) using the levels of satisfaction established by item #14 were significantly different, with F values ranging from 12.2 to 88.8 (p < 0.001 in all cases).

**Conclusion:**

The SATMED-Q was demonstrated to be responsive to different levels of patient satisfaction with therapy in chronically ill subjects. The MID obtained was 13.4 points for the overall normalised scoring scale, and between 10.3 and 20.6 points for domains.

## Background

In recent years, there has been a series of health-related changes in industrialised countries, directly resulting in the introduction of new concepts or factors to be considered when evaluating health care. One of the most important of these changes is the spectacular increase in life expectancy, with the consequent aging of the population. This phenomenon is largely attributable to advances in medicine, and has resulted in changes in mortality and morbidity rates. In the treatment of chronic diseases, the traditional measures of morbidity and mortality, together with other biomedical parameters, only partially evaluate the effectiveness of drugs and other medical interventions which, while prolonging patient life, do not offer a cure. When the treatments administered do not modify survival rates, when there is not a significant difference between them, or when the treatments and other medical interventions cause serious side effects for months or even years, the need arises to evaluate effectiveness in other terms [1]. Health Outcomes Research, a relatively new discipline, focuses among other things on the measurement of disease and treatment impact upon patient-perceived health [2,3].

Patient satisfaction is related to all aspects of healthcare that are of relevance to health. The concept includes satisfaction with both the medical care received and with the specific treatments prescribed by clinicians [4,5]. Patient satisfaction can be considered a pyramid where satisfaction with healthcare is located at the base. This covers all aspects of the care received and includes patient satisfaction with access to medical care, physician behaviour and technical competence, the services provided, the costs, and the treatment selected. Overall treatment satisfaction lies at an intermediate level of the pyramid and includes all related aspects: effectiveness, convenience, undesirable effects, follow-up, etc. Lastly, we find satisfaction with the medication received - this being the patient's evaluation of the process of administering the medication - and the associated results at the top of the pyramid [5]. There would seem to be a relationship between satisfaction with medication and medical treatment and patient adherence or compliance with treatment. It is therefore a quality indicator that can be used to improve healthcare and one that also affects patient preferences [4,6-11]. In addition, knowledge of the degree of satisfaction with treatment may make it easier to predict treatment compliance and help clinicians take health-related decisions. Therefore, this parameter may be a useful indicator to measure in daily practice and biomedical research [12].

The Treatment Satisfaction with Medicines Questionnaire (SATMED-Q) is a brief, feasible and easy to self-administer multidimensional generic questionnaire with good metric properties of reliability and validity [13]. It was designed for use in patients with any chronic disease treated with medicines. The questionnaire was developed assuming the Classical Test Theory framework [14-16], whose properties have been proven to be valid and reliable for chronic health conditions in routine medical practice. Minimal important difference (MID) is a phrase with instant appeal in a field struggling to interpret health-related quality of life and other patient-reported outcomes. It is defined as "the smallest difference in score in the domain of interest which patients perceive as beneficial and which would mandate, in the absence of troublesome side effects and excessive cost, a change in the patient's management" [17]. The terminology can be confusing, with several terms differing only slightly in definition (e.g. minimal clinically important difference, clinically important difference, minimally detectable difference, subjectively significant difference). Four methods are commonly used to estimate MIDs: patient rating of change (global transition items); clinical anchors; standard error (SE) of measurement; and effect size [17,18]. This is important since the MID allows clinicians to better interpret their medical interventions and possible changes in a patient's well-being after treatment with medicines [18]. However, the minimally important difference (MID) of the instrument is as yet unknown. Thus, the aim of this research was to ascertain the MID for the SATMED-Q ? for both its total score and domains.

## Methods

### Design of the study and sample

A multi-centre, cross-sectional, observational study was conducted under routine clinical practice conditions in terms of disease treatment. The study protocol was approved by the Universidad Autónoma de Madrid Independent Ethics Committee (CEI 13-226 on 13 July 2005). The sample of patients analysed for testing the cross-sectional properties of the instrument was also used to calculate MID and effect sizes. The sample design and sampling procedure have been documented elsewhere [13], and sample characteristics are shown in Table 1. In brief, this sample consisted of patients diagnosed with different diseases prevalent in our clinical setting, such as type 2 diabetes mellitus, hypertension, osteoarthritis, benign prostatic hyperplasia (BPH), chronic obstructive pulmonary disease (COPD)/asthma, depression, and migraine. For patient recruitment, the researchers carried out probabilistic sampling at six health centres in the Madrid region and a tertiary hospital in the city of Madrid. Patients were selected from those visiting the centre who met the following study inclusion criteria: male or female outpatients over 18 years of age; diagnosed with one of the aforementioned diseases or health conditions; a duration of treatment for the disorder greater than 2 months at time of enrolment; ability to understand and answer the Spanish versions of the study health questionnaires; and willingness to sign the informed consent form.

**Table 1:** Demographic characteristics of patients included in the study (n = 456)

Age: mean (SD)	62.07 (13.61)			
Gender, male: n (%)	229 (50.2%)			
BMI (kg/m2): mean (SD)	27.77 (4.71)			
Race				
Caucasian	443 (97.2%)			
African	6 (1.3%)			
Other	7 (1.5%)			

Education				
No secondary school	247 (54.2%)			
Completed secondary school	106 (23.2%)			
Vocational training diploma	42 (9.2%)			
University graduate	54 (11.8%)			
Unknown	7 (1.5%)			

		Male	Female	
No. of patients by disease*	< 65 years	≥ 65 years	< 65 years	≥ 65 years
Diabetes	17 (3.7%)	16 (3.5%)	7 (3.7%)	21 (4.6%)
Hypertension	21 (4.6%)	27 (5.9%)	27 (5.9%)	27 (5.9%)
Osteoarthritis	17 (3.7%)	18 (3.9%)	17 (3.7%)	18 (3.9%)
BPH	16 (3.5%)	24 (5.3%)		- -
COPD/asthma	13 (2.9%)	16 (3.5%)	15 (3.5%)	15 (3.5%)
Depression	15 (3.3%)	14 (3.1%)	24 (5.3%)	17 (3.7%)
Migraine	10 (2.2%)	5 (1.1%)	19 (4.2%)	9 (2.0%)
Total	109 (23.9%)	120 (26.4%)	119 (26.2%)	107 (23.5%)

BMI = Body Mass Index; COPD = Chronic Obstructive Pulmonary Disease; BPH = Benign Prostatic Hyperplasia. *Age was missing for one patient.

The size of the sample was determined based on the Rummel's criterion [19], whereby the ratio of subjects to variables should be no less than 4:1. However, the sample size was increased to allow statistical comparisons between meaningful groups related to the validation study. Thus, to prevent missed data, we decided to select a minimum of 50 patients (25 males and 25 females) corresponding to each of the 7 disease conditions considered in the study, representing a minimum of 350 patients in total. The validation sample finally comprised 456 patients. Table 1 shows the number of cases sampled per stratum, in addition to the mean age, mean body mass index (BMI), and the distributions for ethnicity and educational level.

### Scales

In addition to the SATMED-Q, the Spanish version of the TSQM (Treatment Satisfaction Questionnaire for Medication) [20] was used to test concurrent validity of the SATMED-Q, and item #14 of the scale in particular was also used as an anchor reference. The TSQM is a 14-item Likert-type self-administered instrument with 4 subscales or domains: satisfaction with side-effects, effectiveness, convenience of use and overall satisfaction, which account for 77% of available variance. Moreover, it is possible to calculate a total composite score summarising all domains.

As mentioned above, the SATMED-Q is a brief, feasible and easy to self-administer multidimensional generic questionnaire comprising 17 Likert-type items [13]. It has been designed for use in patients with any chronic disease taking any type of prolonged pharmacological treatment. The instrument is made up of six domains or dimensions exploring satisfaction with drug efficacy, side-effects, convenience of use, medical care, impact on activities of daily living and general satisfaction, which account for 80.8% of available variance. It also provides an overall score for satisfaction with drug treatment by summing up all ?domains.

Both scales were self-administered in the waiting room during the same office visit. Item #14 of the TSQM (Taking all things into account, how satisfied or dissatisfied are you with this medication?) was used as an anchor reference since it directly explores satisfaction with drug treatment on a seven-category ordinal scale, from extremely satisfied to extremely dissatisfied, with a neutral category in the middle.

### Calculation of the Minimally Important Difference (MID) and statistical procedures

A triangulation approach was followed to estimate the MID of the SATMED-Q: an anchor-based method and three distribution-based methods [21]. The mean absolute differences in total score between the neutral category response and the contiguous satisfied/dissatisfied response categories (both merged into a single response group) for TSQM item #14 were considered a valid estimate of MID values for the overall scoring of the SATMED-Q instrument using an anchor-based approach. A similar procedure was followed to obtain MID estimates for each individual SATMED-Q dimension. Patients were first classified into four categories according to responses to item #14 in order to quantify the magnitude of the patient's perceived distance from the neutral response category: extremely satisfied/dissatisfied (merged), very satisfied/dissatisfied (merged), satisfied/dissatisfied (merged), neither satisfied nor dissatisfied (single neutral category). Average score and standard deviation were computed for each response group for the total score and for each domain of the SATMED-Q expressed as a 0-100 normalised or standardised score. This methodological approach has previously been used by other researchers [17,21-26]. Although our main concern was with the minimal perceived difference, differences for all response groups with respect to the neutral category were also computed since we found step-scaled differences to be informative, and we did not know in advance if perceived differences could be monotonically distributed by corresponding distance levels.

Difference between neutral and satisfied/dissatisfied was considered to be the MID value, while differences between neutral and the other two categories were classified as medium and large differences, respectively.

Effect size (ES) was also computed as a distribution-based method along with the standard error of measurement (SEM) and one-half of a standard deviation (SD) to support congruence of the main method for MID calculation, as other investigators have done in the field of patient satisfaction with medical care [25,26]. The ES was computed according to the Kazis et al. method [27], dividing the SATMED-Q mean response difference for the minimum change level determined using TSQM item #14 by the pooled standard deviation of the whole sample for a given domain or the total score. Interpretation of the effect size was based on the established criterion which considers an effect size of 0.20 - <0.50 as small; a size of ≥ 0.50 and < 0.80 as moderate; and a size of ≥ 0.80 as large [27]. The SEM was obtained by multiplying the baseline standard deviation of the scale, or domain, by the square root of one minus its reliability coefficient [28]. Scale reliability was estimated using Cronbach's alpha coefficient [29].

In addition to the above analyses, exploratory descriptive statistics were performed using measures of central tendency and dispersion, and the Kolmogorov-Smirnov test was applied to test for a normal distribution of scores. An ANOVA and the Levene test for homogeneity of variance testing were used to check that differences in SATMED-Q scores could be interpretable when the sample was stratified by satisfaction levels using TSQM item #14.

A bootstrap re-sampling method was used to obtain confidence interval estimates for the MID and medium and large differences. A total of 1000 random samples with replacement were extracted and mean differences from the reference category were computed. The percentile method was used to obtain 95% confidence limits. Bootstrap estimates may also be considered a more valid measure of general population values when clinical samples are used and community samples are not gathered, given that they increase the likelihood of identifying outlier subjects to be re-sampled.

Since both TSQM and SATMED-Q are patient reported outcomes (PRO), it could be argued that there might be a lack of external validity in the assessment of minimally important differences. For this reason, differences in the SATMED-Q effectiveness dimension were compared with treatment effectiveness groups as assessed by the clinician on a 4-point Likert-type scale (poor, acceptable, good, and excellent). Mean difference values are reported as a reference.

All tests were two-sided and a type I error (α) < 0.05 was assumed to be significant. A Bonferroni adjustment was applied for multiple comparisons. All analyses were performed using SPSS version 18.0 statistical software.

## Results

The cross-sectional phase of the study for the development of the SATMED-Q enrolled 456 subjects with different diseases or health conditions (Table 1). The item non-response rate was very low: 96.7% of the patients answered all questions on the questionnaire. The average response time was 4.71 minutes (SD = 4.65). The median completion time was 4 minutes. The overall composite scores exhibited a negative skewed distribution, with a mean of 75.03 and a standard deviation of 14.76 on the 0-100 standardised scale. The median value was 77.08. The minimum recorded score was 17.36 and the maximum was 100. Individual item response distribution covered all proposed response categories for all items, although no individual simultaneously selected the minimum score for all items in the scale. With the exception of the *undesirable side effects *subscale, the distribution of responses showed a slight negative skewness; the item with the most skewed distribution (*willingness to continue treatment*) included 44% of the responses in the upper part of the scale. All distributions were unimodal. The *undesirable side effects *subscale accumulated responses in the lower portion of the scale; between 66% and 75% of the responses were located in the category "No, not at all".

The SATMED-Q total and ?domain scores significantly correlated with the total and domain scores on the Spanish version of the TSQM (Table 2). A correlation of 0.74 was obtained between the composite scores for both scales, with correlations ranging from 0.58 to 0.68 between dimensions having similar contents (*p *< 0.001 in all cases). A significant relationship (p < 0.01) was also found between the scores on the SATMED-Q and item #14 of the TSQM, ranging from 0.18 to 0.58. The correlation between the medical care domain of the SATMED-Q and item #14 of the TSQM, although statistically significant, was weak (r = 0.18). However, the other domains showed moderate-to-good correlation coefficients, supporting the validity of this item as an anchor for calculating MID for the SATMED-Q tool.

**Table 2:** Pearson correlation coefficients between SATMED-Q domains, TSQM domains and TSQM item #14

			TSQM			
	
SATMED-Q	Effectiveness	Side effects	Convenience of use	General satisfaction	Total score	Item #14
Treatment effectiveness	0.68	0.19	0.32	0.67	0.61	0.55
Convenience of use	0.32	0.34	0.68	0.37	0.56	0.34
Impact on daily living/activities	0.44	0.19	0.28	0.55	0.50	0.45
Medical care	0.19	0.02NS	0.10**	0.28	0.19	0.18
Undesirable side effects	0.18	0.58	0.24	0.25	0.43	0.23
General satisfaction	0.60	0.27	0.41	0.68	0.66	0.58
Overall composite score	0.61	0.39	0.51	0.70	0.74	0.58

*p *< 0.001 in all cases, except: ** *p *< 0.01 and NS = Non-significant. TSQM = Treatment Satisfaction Questionnaire for Medications. SATMED-Q = Satisfaction with Medicines Questionnaire.

**Figure 1:**
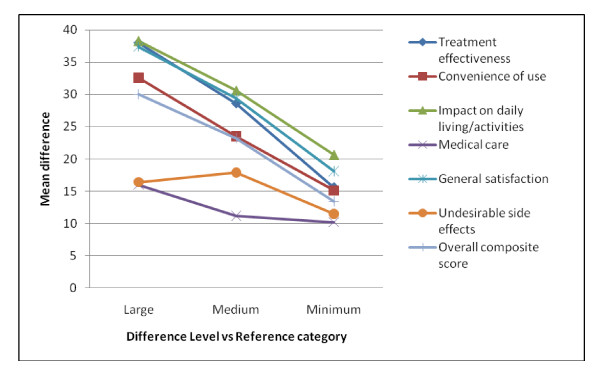
**Mean SATMED-Q differences by TSQM item #14 difference level with respect to reference category and by dimension**.

When using TSQM item #14 to scale perceived differences in satisfaction, statistically significant SATMED-Q mean differences were observed at each level of difference in satisfaction (minimum, medium and large) as compared with the reference (neutral) category (see Table 3), meaning that the classification of different levels of patient satisfaction by means of TSQM item #14 is valid and appropriate for estimating the MID with such item as an anchor-based method, as has previously been applied by others [25]. Observed difference sizes versus the reference category were monotonous and almost linear for the SATMED-Q total score and for the separate dimensions, except for the undesired side effects dimension, where a small decrease in the size of differences versus the reference category was observed for the large difference level (Figure 1), which could be due to the small sample size in this group (39 subjects with only four responding extremely dissatisfied).

**Table 3:** Standardised scores for SATMED-Q domains and total score by magnitude of difference in response categories of TSQM item #14

Domain	Difference	N	Mean	SD	F (p value)
	Large	39	89.5	12.9	58.1 (p < 0.001)
Treatment effectiveness	Moderate	136	80.1	18.2	
	Small	213	67.1	19.9	
	Reference	68	51.5	20.8	

	Large	39	90.4	15.1	26.3 (p < 0.001)
Convenience of use	Moderate	136	81.3	20.8	
	Small	213	72.9	21.1	
	Reference	68	57.8	26.3	

	Large	39	81.8	18.9	40.4 (p < 0.001)
Impact on daily	Moderate	136	74.1	22.2	
living/activities	Small	213	64.1	22.3	
	Reference	68	43.5	21.2	

	Large	39	85.9	21.5	10.7 (p < 0.001)
Medical care	Moderate	136	81.1	18.7	
	Small	213	80.1	17.8	
	Reference	68	69.9	22.3	

	Large	39	94.5	9.7	60.4 (p < 0.001)
General satisfaction	Moderate	136	86.5	17.4	
	Small	213	75.2	19.0	
	Reference	68	57.1	22.2	

	Large	39	92.9	15.2	12.2 (p < 0.001)
Undesirable	Moderate	136	94.4	13.8	
side effects	Small	213	88.0	20.8	
	Reference	68	76.5	24.4	

	Large	39	89.3	8.7	88.8 (p < 0.001)
Composite score	Moderate	136	82.5	11.3	
	Small	213	72.7	13.0	
	Reference	68	59.3	12.1	

Values are means and SD (standard deviation). TSQM = Treatment Satisfaction Questionnaire for Medications. SATMED-Q = Satisfaction with Medicines Questionnaire.

Mean SATMED-Q scores (total and domain) in the TSQM item #14 neutral satisfaction category and in the satisfied/dissatisfied category are shown in Table 4. The raw difference between the two mean scores represents the estimated MID. MID estimates ranged from 10.3 (satisfaction with medical care domain) to 20.6 (satisfaction with impact of medicines on daily living activities). SEM values ranged from 5.9 (total score and satisfaction with undesirable side effects) to 9.5 points (satisfaction with treatment effectiveness). The magnitude of effect size estimates was moderate to large, ranging from 0.58 (satisfaction with undesirable side effects) to 0.91 (overall satisfaction score). In particular, the effect size estimate for the total score was the largest (0.91), indicating that the estimated MID for the total score (13.4) seems to be a reasonable magnitude for the minimum perceived change in overall satisfaction with drug treatment.

**Table 4:** Minimally Important Difference (MID), standard error of measurement (SEM) and effect size of the standardised SATMED-Q total and domain scores calculated for the sample of 456 patients to test psychometric properties of the instrument

				Distribution-based MID		
					
SATMED-Q domain	SATMED-Q mean score at neutral satisfaction category of TSQM item #14	SATMED-Q mean score at satisfied/dissatisfied category of TSQM item #14	Anchor-based MID^1^	SEM^2^	One-half SD	Effect size^3^
Treatment effectiveness	51.5 (20.8)	67.1 (19.9)	15.6	9.5	10.9	0.71
Convenience of use	57.8 (26.3)	72.9 (21.1)	15.1	8.6	11.6	0.65
Impact on daily living/activities	43.5 (21.3)	64.1 (22.3)	20.6	9.4	12.2	0,85
Medical care	69.9 (22.3)	80.1 (17.8)	10.3	8.1	11.9	0.43
General satisfaction	57.1 (22.2)	75.2 (19.0)	18.2	8.1	10.6	0.83
Undesirable side effects	76.5 (24.4)	88.0 (20.8)	11.5	5.9	10.0	0.58
Total score	59.3 (12.1)	72.7 (13.0)	13.4	5.9	7.4	0.91

Values are means (standard deviation). 1Computed as the raw difference between scores in neutral response and satisfied/dissatisfied categories of TSQM item #14 used as an anchor reference. 2 SEM = Standard error of measurement obtained by multiplying the standard deviation of each domain or the total score in the entire sample by the root square of 1 minus the coefficient of reliability (-Cronbach's α). 3Obtained by dividing the MID of the domain or total score by the standard deviation of the whole sample for that domain or total score. TSQM = Treatment Satisfaction Questionnaire for Medications. SATMED-Q = Satisfaction with Medicines Questionnaire. SD = standard deviation.

Table 5 contains bootstrap estimates for MID for each dimension and total scores for the SATMED-Q. Bootstrap mean values for 1000 samples were close to the asymptotic theory estimates, with the exception of the satisfaction with medical care dimension. Mean MID for this dimension was close to 0 and the 95% confidence interval included the null difference value, ranging from negative to positive values. However, the difference for the contiguous level of satisfaction (medium difference) did detect positive differences (mean MID = 10.13, SE = 3.13).

**Table 5:** Bootstrap MID estimates for 1000 samples

SATMED-Q domain	Mean MID	SE	2.5 Percentile	97.5 Percentile
Treatment effectiveness	16.02	2.84	10.54	21.46
Convenience of use	15.29	3.51	8.15	22.04
Impact on daily living/activities	21.07	2.98	14.99	26.91
Medical care	-0.21	3.31	-6.68	6.47
General satisfaction	18.84	2.94	13.00	24.33
Undesirable side effects	11.44	3.41	4.66	18.30
Overall score	13.64	1.72	10.26	17.10

SATMED-Q = Satisfaction with Medicines Questionnaire. MID = Minimum Important Difference. SE = standard error.

When comparing effectiveness groups as assessed by the clinician, differences in mean values for the SATMED-Q dimension corresponding to satisfaction with treatment effectiveness attained significance. Taking the Acceptable Effectiveness group as the reference, the difference with the Poor Effectiveness group was the largest (d = -23.4, SE = 5.78, p < 0.001), differences with Good Effectiveness did not attain significance (d = 6.26, SE = 2.54, p = 0.066), and the difference with Excellent Effectiveness was also large (d = 18.76, SE = 2.76, p < 0.001).

## Discussion

It is increasingly recognised that the patient viewpoint should be taken into account when evaluating a medical treatment. One domain of such a patient-oriented evaluation is patient satisfaction with treatment or treatment satisfaction. Treatment satisfaction is a documented area of interest within health outcomes research and appears to be increasingly used as a patient-reported outcome when testing new or existing treatments [30]. Patient satisfaction with the medication received is of growing concern in clinical practice. On the one hand, this is because satisfaction helps evaluate the benefits and convenience of the medication provided. On the other hand, the fact that treatment satisfaction is associated with increased patient adherence to therapy and to a greater patient desire to continue using the drug may help predict treatment compliance and improve effectiveness of the administered therapy [31,32], with closer follow-up of those patients expected to adhere less to treatment. The aim of this study was to determine the minimally important difference (MID) for the recently available SATMED-Q, an instrument for exploring patient satisfaction with treatment for a medicine on a generic basis.

The MID found in this study shows that a change within the range of 10.26-17.10 points in the total score (on a scale of 0-100 points) would be required for a change to be detectable by the patient in his/her level of satisfaction with drug treatment, i.e. for a modification in the treatment to be meaningful from the patient's perspective. Moreover, this noticeable change should therefore be clinically meaningful for physicians in order to help them take the appropriate health decision, such as whether to continue the therapy or change it, depending on the patient response. The MID for the questionnaire domains ranged from 10.3 to 20.6. These values could be considered relevant as they showed a moderate-to-large effect size [27], meaning that patients should be able to detect a change in drug treatment when this actually happens.

However, the MID values detected were approximately equivalent to 1.96 times the value of the corresponding SEM figures; almost double or more that which has been interpreted previously as equivalent by other authors also using this distribution-based method [25,26,33-35]. The explanation for this discrepancy could be that, compared with other instruments assessing quality of life, patient preference, etc., satisfaction with medicinal treatment needs a larger difference in scores to be detectable by the patient. Due to the subjacent or latent construct of satisfaction, this could even be expected. For example, during the development of the PASAPQ questionnaire [25], Kozma et al. also found a similar discrepancy in MID estimation between anchor-based and distribution-based methods. In that instrument, MID calculated with an anchor method was between two and three times the value calculated with distribution methods. However, when Vernon et al. [26] developed the MSQ in patients with schizophrenia, they found more congruence in MID estimation using both anchor and distribution-based methods. Part of this lack of congruence could be due to the fact that MSQ is a one-item instrument in comparison with multi-item questionnaires such as the PASAPQ or SATMED-Q. Also, discrepancies could be due to the type of medical interventions explored with such instruments or the ceiling effect that these patient satisfaction instruments may have by the very nature of this construct or by the response categories used for the items [5]. Interestingly, further research should clarify whether current methods remain useful for triangulating the estimation of the MID value of a PRO instrument or whether researchers would need new approaches, particularly in the field of patient satisfaction. In fact, we could not apply an anchor-based method other than the one used here mainly because this study was cross-sectional and there was only one visit (i.e. a scale measuring change in patient satisfaction could not be administered).

Two methodological issues should be taken into account. Firstly, each dimension consists of 3 items on a 5-point Likert-type scale (except satisfaction with medical care, which contains only 2 items). Hence, raw dimension scores will range from 0 to 20 and a 1-point change will translate to less than 7 points on the 0-100 standardised scale, and a change of 10 points for the satisfaction with medical care dimension. This being so, a change of 10 to 20 points will be quite easy to accomplish from the moment when patient scores vary. On the other hand, satisfaction scores are typically negatively skewed, and deviations from the main bulk of scores usually reflect a large change.

As we have seen, results are less conclusive for the satisfaction with medical care dimension. Differences between adjacent satisfaction groups are difficult to distinguish according to bootstrap estimates and larger changes need to be made in order for a patient to recognise a meaningful change.

We find that variations greater than 6 points are needed in order to consider a change in patient health situation to be valid, at least as regards the satisfaction with treatment effectiveness dimension. Nevertheless, we cannot rule out other reasons explaining the range of MID values obtained, such as the methods used here to calculate the MID, which could be taken as a limitation of this research. We were not able to use the typical prospective approach of measuring a change with an external scale as the anchor for MID calculation in quality-of-life instruments, e,g, the Juniper et al. patient global assessment scale [36-38]. Instead we used the ability of the instrument to distinguish between the two nearest levels of satisfaction, starting with a neutral category where the subjects are unable to determine their level of satisfaction. Another possible limitation is the calculation of the sample size, which was established in the original research by testing the psychometric properties of the SATMED-Q, and not calculated for the MID.

Possible implications of the MID value of SATMED-Q, from a clinical standpoint, still need to be established. However, as mentioned previously, it may be clinically meaningful for physicians in order to help them take the appropriate health decision in the therapeutic management of patients. Other implications of MID could be the use of this value to classify patient responders/non-responders to treatment with medicines or also for sample size calculation in clinical and/or observational trials. For future prospects, this MID value should be tested to explore its ability to correlate with effectiveness of therapy and patient compliance with treatment, since this could not be explored due to the design of our study.

## Conclusion

Taking into account the above limitations, the SATMED-Q was demonstrated to be responsive to different levels of patient satisfaction with therapy in chronically ill subjects. MID values for the instrument are now available, allowing researchers to use it to determine sample sizes for studies based on patient satisfaction outcomes and/or as a measurement of effectiveness in studies with end-points based on patient perspectives.

## Authors' Disclosure

## Competing interests

Javier Rejas and Javier Soto are employees of Pfizer, S.L.U. Miguel Ruiz and Antonio Pardo are professors of psychology at the School of Psychology of the Universidad Autonoma de Madrid. All authors declare they have no competing financial or non-financial interests as a consequence of this work.

## Authors' contributions

This was a collaborative project and the authors worked closely together. All participated in the design of the original study and in the interpretation of data and drafting of the manuscript. JR and MR were also responsible for the statistical analysis. All the authors were responsible for the review of the literature and extraction of references. All the authors read and approved the final manuscript.

## Pre-publication history

The pre-publication history for this paper can be accessed here:

http://www.biomedcentral.com/1471-2288/11/142/prepub
